# Mechanical Thrombectomy for Submassive Pulmonary Embolism in an Adult with Duodenal Adenocarcinoma and Brain Metastases

**DOI:** 10.7759/cureus.6072

**Published:** 2019-11-04

**Authors:** Mark N Alshak, Justin M Lebenthal, Anne Covey, Chhavi Kumar

**Affiliations:** 1 Medicine, Weill Cornell Medical College, New York, USA; 2 Internal Medicine, New York-Presbyterian/Weill Cornell Medical Center, New York, USA; 3 Interventional Radiology, Memorial Sloan Kettering Cancer Center, New York, USA; 4 Hospital Medicine, Memorial Sloan Kettering Cancer Center, New York, USA

**Keywords:** pulmonary embolus, mechanical thrombectomy, duodenal adenocarcinoma, brain mets, submassive pe, hospitalist

## Abstract

A 52-year-old African American man with small bowel adenocarcinoma metastatic to the brain and leptomeninges was found to have an acute pulmonary embolism while hospitalized for acute lower limb weakness, bowel, and urinary incontinence. He had an elevated troponin, echocardiographic findings concerning for right heart failure, and bilateral segmental and subsegmental pulmonary artery perfusion deficits with right pulmonary artery thrombus on CT angiography. Given the laboratory and radiographic findings with normotensive blood pressure, the patient was diagnosed with a submassive pulmonary embolism.

After deliberation with interventional radiology and hematology, the patient underwent mechanical thrombectomy and was treated with therapeutic anticoagulation. Mechanical thrombectomy revealed substantial clot burden in the central pulmonary arteries and yielded a significant improvement in hypoxia and dyspnea.

This case was an excellent exercise in therapeutic decision-making amidst a dynamic disease process that required integration of numerous clinical and diagnostic data points, including empiric anticoagulation with high clinical suspicion of acute pulmonary embolism, review of contraindications to anticoagulation and thrombolysis in metastatic malignancy, and the decision to pursue mechanical thrombectomy in the setting of contraindications to catheter-directed thrombolysis.

## Introduction

Patients with active malignancy have a 3-5-fold higher risk of thromboembolism with an occurrence rate of approximately 15% [1]. Moreover, gastrointestinal (GI) malignancies have almost a 3 times higher risk of venous thromboembolism as compared to their non-GI malignancy counterparts [2]. Pulmonary embolism (PE) is estimated as the second leading cause of death in patients with active malignancy [1]. If left untreated, acute PE can have a mortality rate of up to 30% [3]. Cardiopulmonary decompensation can occur within the first few hours of presentation and as such it is prudent for clinicians to act quickly when PE is suspected [3]. We present a case of a man with duodenal adenocarcinoma metastatic to the brain and leptomeninges who was found to have an acute pulmonary embolism while hospitalized for acute lower limb weakness and incontinence after having prophylactic anticoagulation held due to traumatic hematuria following catheter placement. 

## Case presentation

A 52-year-old African American man with metastatic small bowel adenocarcinoma and medical history significant for hypertension and sickle cell trait was admitted for the management of asymptomatic hyponatremia and left lower extremity weakness with bowel and bladder incontinence. He had initially presented for outpatient chemotherapy and was sent to the urgent care center after being found to be hyponatremic to 121 mEq/L.

Upon admission, a diagnostic workup for left lower extremity weakness with bladder and bowel incontinence was initiated. Due to the bladder incontinence, a Foley catheter was placed which caused traumatic injury to the urethra. Gross hematuria was noted with blood present at the urethral meatus and his prophylactic low molecular weight heparin was held. We obtained MRI that showed extensive leptomeningeal metastases along the distal thoracic spinal cord, conus medullaris, and nerve roots of the cauda equina. Further evaluation for intracranial involvement was scheduled for the following morning. 

The next morning before his imaging, the patient experienced acute onset dyspnea and right-sided chest pain. On exam, he was acutely tachycardic to 130 beats per minute, tachypneic to 29 respirations per minute, hypertensive to 145/88 mmHg, hypoxic to 85% SpO2 on room air, and endorsed left calf tenderness and warmness without swelling. 

**Figure 1:**
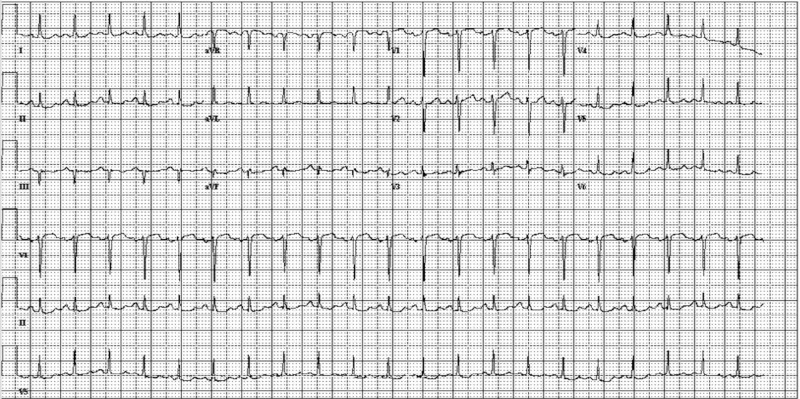
ECG at the Time of Symptom Onset

An ECG was performed to evaluate for primary cardiac etiologies (Figure 1). ECG did not show any changes consistent with an ST-elevation myocardial infarction. EKG revealed ST depression in leads I, II, V4, V5. It also revealed, sinus tachycardia, the most common EKG finding in PE. Serum troponin at that time was negative. 

**Figure 2:**
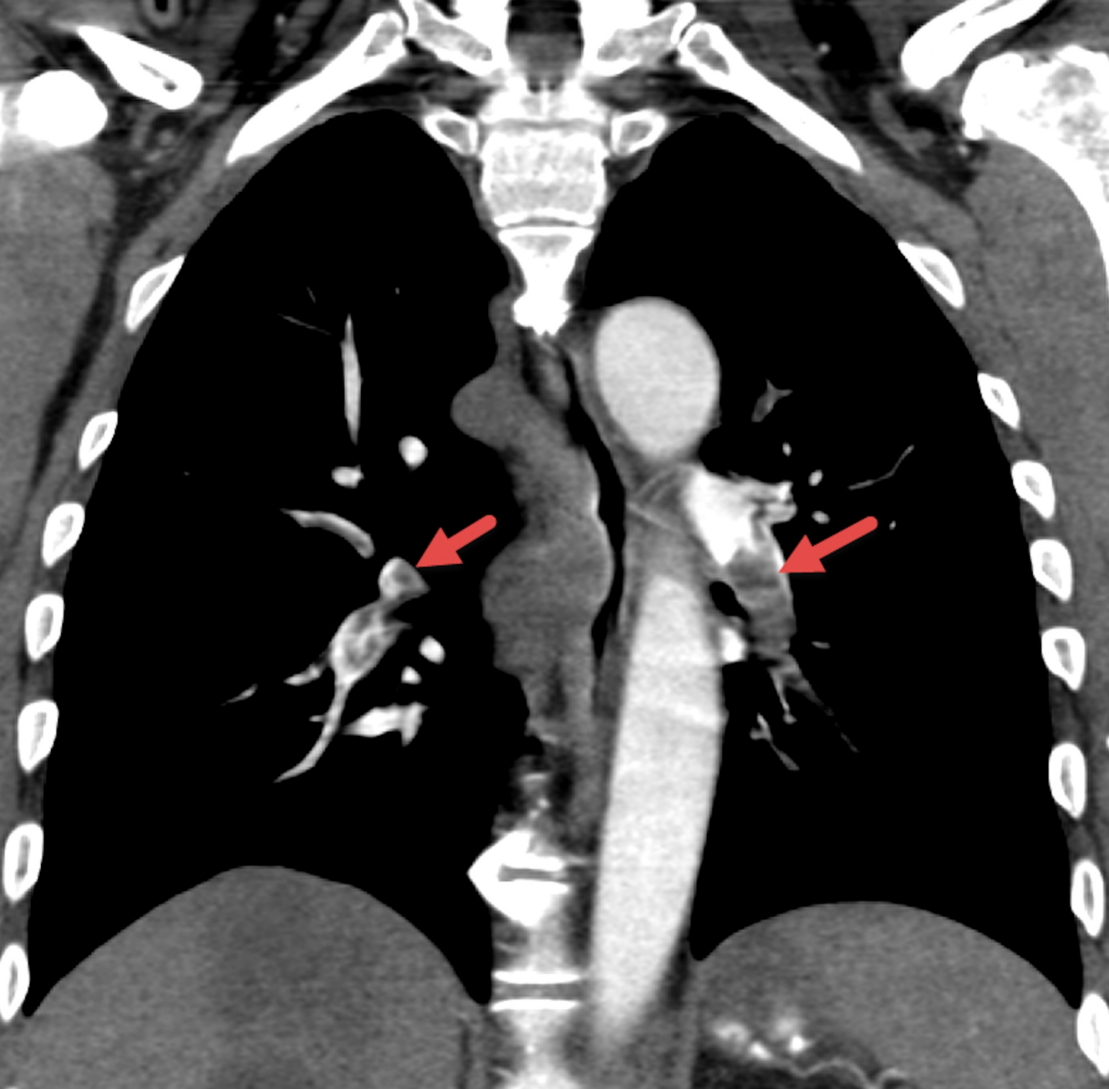
CTPE Obtained Shortly After the Time of Symptom Onset Showing Perfusion Defects

Before any further investigation was conducted, therapeutic low molecular weight heparin (LMWH) was given. Contrast-enhanced CT angiography of the chest demonstrated extensive bilateral pulmonary emboli, including distal main right and left pulmonary arteries, involving lobar, segmental, and subsegmental arteries of all lobes with right ventricular dilatation (Figure 2). 

**Figure 3:**
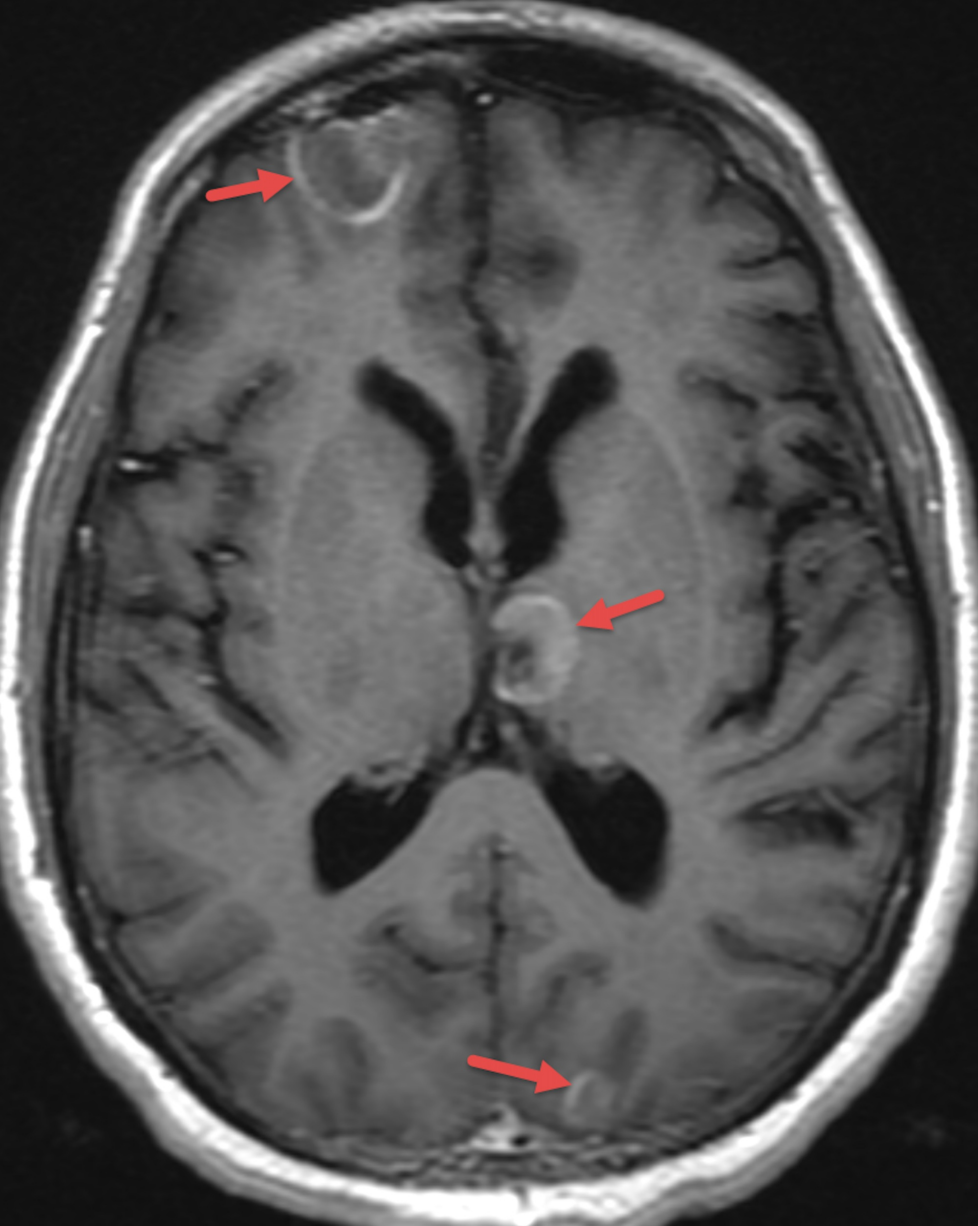
MRI of the Brain Showing Metastatic Disease

Previously scheduled MRI of total spine and brain with and without contrast showed multiple supra and infratentorial leptomeningeal and parenchymal metastases with involvement of the entire spinal cord as well as left posterior medial sixth rib metastasis (Figure 3). It was at the same time that his second troponin level resulted and was elevated to 0.28 ng/mL. 

Echocardiogram was then performed and showed pulmonary hypertension with a pulmonary artery pressure of 44 mmHg with moderate tricuspid regurgitation, right atrial and ventricular enlargement, severe global right ventricular dysfunction, and a positive McConnell’s sign. His third troponin level was elevated to 0.33 ng/mL. He continued to appear distressed with increasing shortness of breath and tachypnea in spite of being on therapeutic anticoagulation.

**Figure 4:**
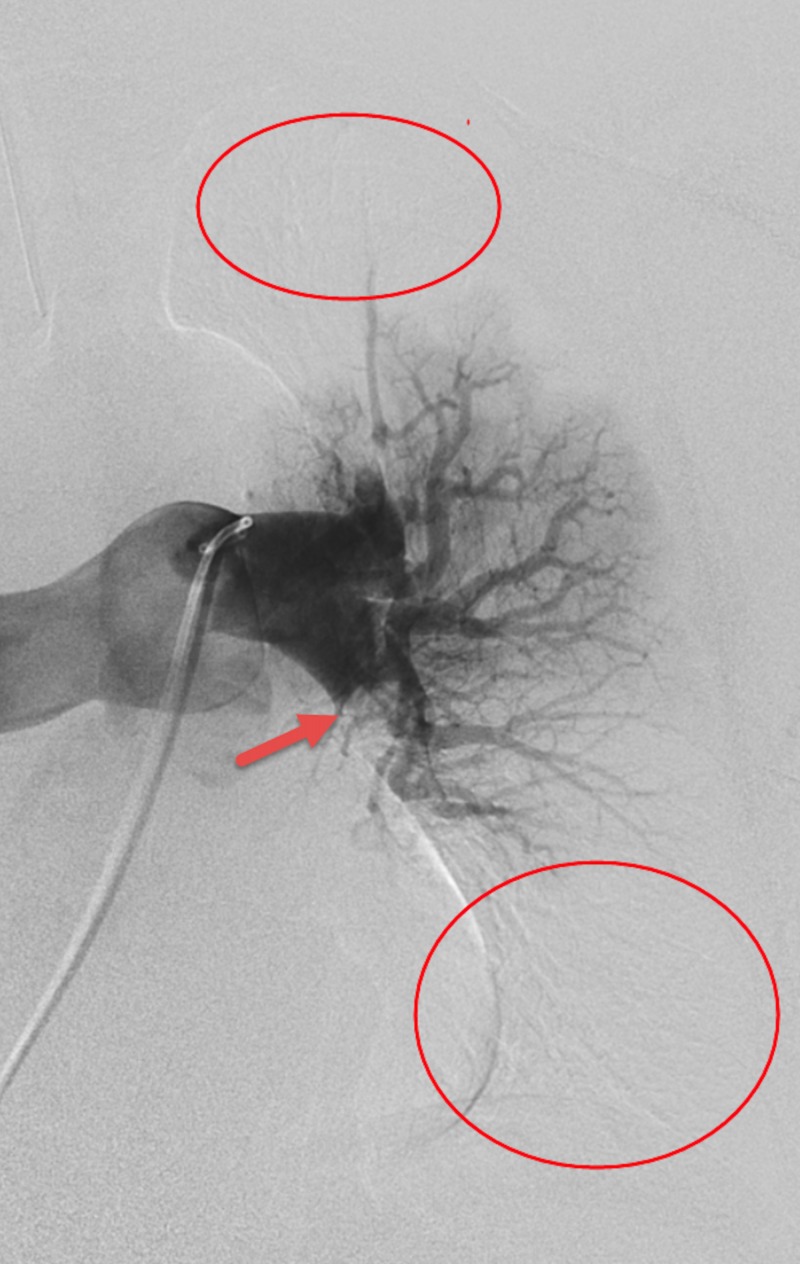
Left Lung Pre-Mechanical Embolectomy CT Pulmonary Angiography Showing Absence of Perfusion to Large Areas of the Upper and Lower Lobes

**Figure 5:**
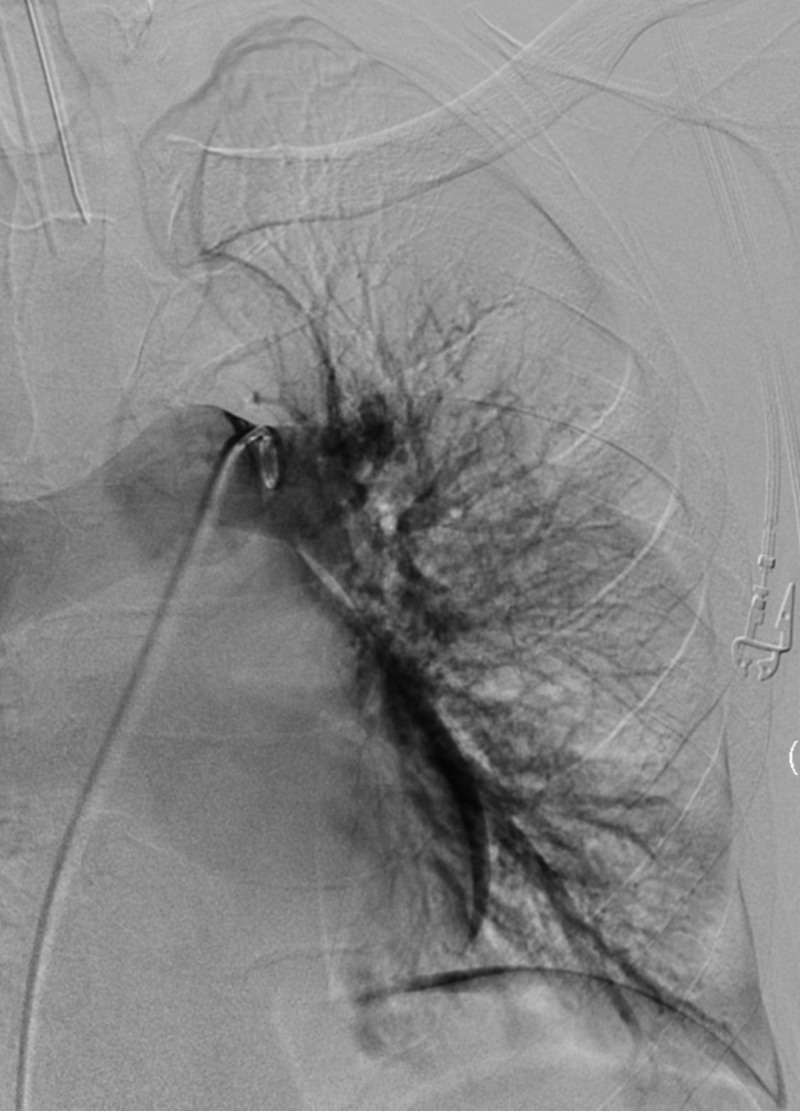
Left Lung Post-Mechanical Embolectomy CT Angiography Showing Improved Perfusion to the Upper and Lower Lobes

**Figure 6:**
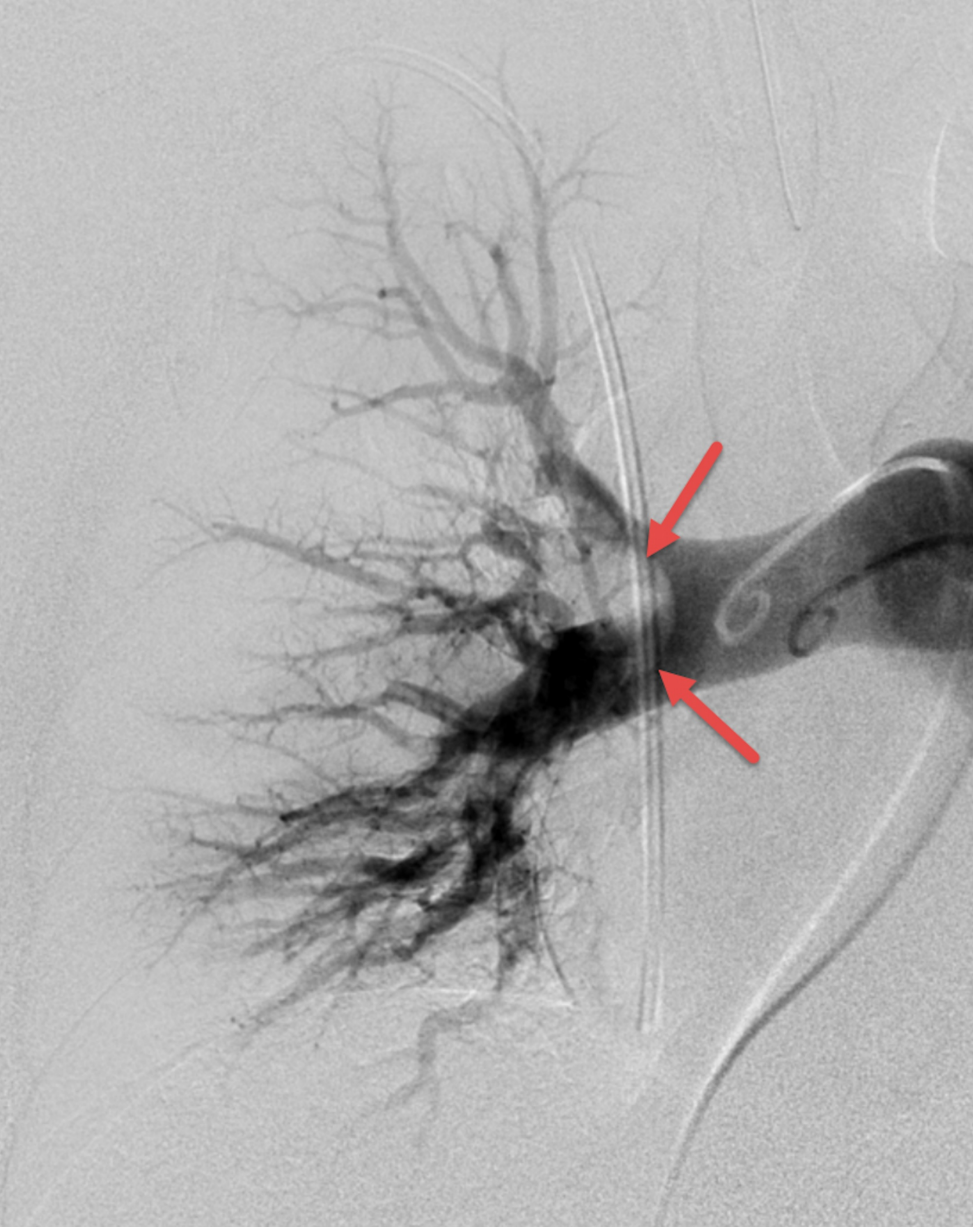
Right Lung Pre-Mechanical Embolectomy CT Angiography Showing the Meniscus of the Large Embolus in the Lower Lobe.

**Figure 7:**
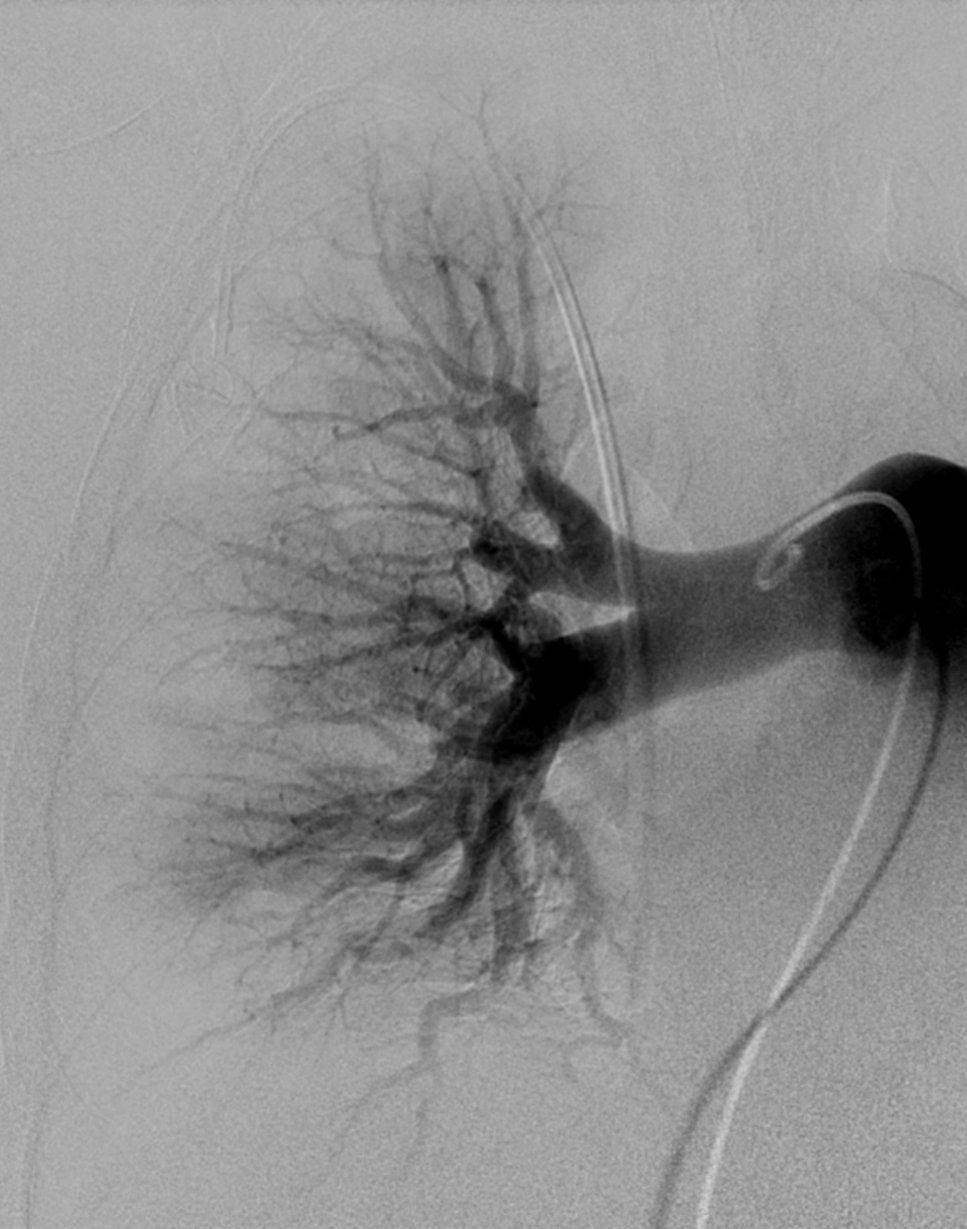
Right Lung Post-Mechanical Embolectomy CT Angiography

**Figure 8:**
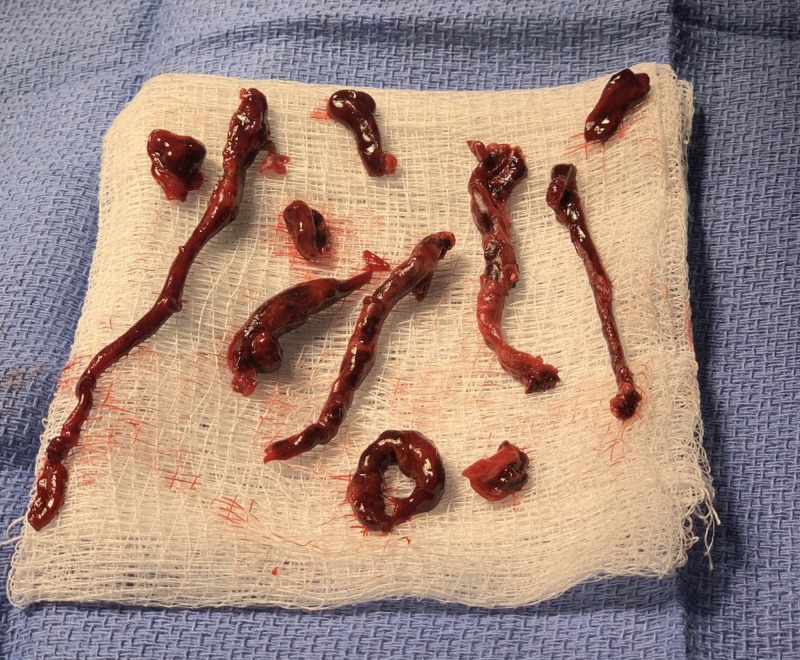
Gross Image of Pulmonary Emboli Post-Mechanical Thrombectomy

Because of the traumatic hematuria secondary to a Foley catheter insertion attempt, DVT prophylaxis was held for a total of 36 hours prior to the initiation of LMWH for suspected PE. Empiric anticoagulation with LMWH was initiated within an hour after the patient’s acute change in clinical status given the high index of suspicion for pulmonary embolism as well as his high Geneva score. Anticoagulation was continued despite discovering significant intracranial burden of disease after deliberation with the hematology service. However, given the concern for intracranial hemorrhage with catheter-assisted thrombolysis, an extensive conversation with interventional radiology occurred and the decision was made to pursue mechanical thrombectomy. Multiple large clots were removed from his segmental and subsegmental pulmonary arteries (Figures 4-8). 

After mechanical thrombectomy, he significantly improved symptomatically and hemodynamically. He was continued on therapeutic LMWH to avoid further progression of thrombosis. As his underlying malignancy progressed, he developed a cranial nerve III palsy which prompted a CT scan of his head to assess for hemorrhage in the setting of therapeutic anticoagulation. He subsequently had three CT scans of his head which all showed no active or previous hemorrhage and his cranial nerve III palsy was attributed to his known intracranial lesions. 

## Discussion

Patients with active malignancy, especially GI cancers, have a much higher risk of thromboembolism [1, 2]. Given this heightened risk of DVT, clinicians should have a high threshold for withholding prophylactic anticoagulation in hospitalized, immobilized patients with active malignancy. Indications for withholding anticoagulation prophylaxis include, but are not limited to, active clinically significant bleeding, severe thrombocytopenia (<50,000/ μL), major trauma, obstetric delivery, previous intracranial hemorrhage, intracranial or spinal tumor, neuraxial anesthesia, and severe hypertension [4]. If the decision is made to withhold prophylactic anticoagulation, this decision should be routinely reconsidered on a daily basis. 

PE is estimated as the second leading cause of death in patients with cancer with rates of up to 30% mortality if left untreated [1, 3]. Quick recognition and action are prudent when PE is suspected as cardiopulmonary decompensation can occur within the first few hours of presentation. Given our high index of clinical suspicion and the patient’s elevated risk for decompensation, we felt empiric therapeutic anticoagulation before confirmation was appropriate even in the setting of possible intracranial metastases [5]. Intracranial tumors are a relative contraindication to anticoagulation given the high risk of intracranial hemorrhage. Several studies have sought to characterize which solid tumor types are at an increased risk of hemorrhage. However, because of the high risk of hemorrhage and the resulting morbidity and mortality, patients with brain lesions are rarely enrolled in randomized anticoagulation therapy trials [6]. Donato et al. showed that metastatic melanoma and renal cell carcinoma were at a 4-fold higher risk of intracranial hemorrhage as compared to metastatic lung cancer, although treatment with LMWH did not influence the rates of hemorrhage in either the treated or untreated group [7]. This data would suggest that anticoagulation does not increase the risk of intracranial hemorrhage in patients with brain metastasis. However, the small data from which this retrospective study derives its findings warrants larger, multi-center prospective studies to validate their conclusions. Moreover, it is important for the provider to conduct a risk-benefit analysis of their patients considering their medical history, relative and absolute contraindications, type of malignancy, and balance with the risk of cardiopulmonary collapse due to untreated pulmonary embolism. 

This patient met the criteria for a submassive PE based on positive troponin levels and echocardiographic findings suggestive of right heart strain. Pulmonary embolism is divided into three categories: massive, submassive, and low risk. Patients with massive PE have hemodynamic compromise defined as systolic blood pressure <90 mm hg or a drop in systolic blood pressure of at least 40 mm Hg, while a submassive PE has evidence of right ventricular dysfunction with normal blood pressure [8]. Low-risk PE has no evidence of right ventricle dysfunction [8]. The varying classifications are important as it dictates the treatment options available. Treatment options for submassive PE include thrombolysis, catheter-assisted embolectomy, surgical embolectomy, anticoagulation, and placement of an IVC filter [9]. Current guidelines lack the data to support the routine use of advanced interventions, such as embolectomy, in intermediate-to-high risk submassive PE and the decision to use more advanced therapies needs to be made on a case-by-case basis [10,11]. In cases where thrombolysis is absolutely contraindicated, such as intracranial neoplasm, mechanical thrombectomy is a reasonable alternative [12]. Further, absolute contraindications of thrombolysis include acute intracranial hemorrhage, history of intracranial hemorrhage, severe uncontrolled hypertension, serious head trauma or stroke within the past 3 months, thrombocytopenia and coagulopathy, therapeutic dose of LMWH within the past 24 hours, direct thrombin inhibitors, factor Xa inhibitors, and severe hypo- or hyperglycemia [10]. Submassive PE cases constitute 31% of diagnosed pulmonary embolisms and have in-hospital mortality of 5-12.6% [13,14]. Even though thrombolysis is first-line therapy, the high mortality associated with submassive PE drove us to provide immediate treatment. 

## Conclusions

A careful risk-benefit analysis should precede the decision to hold prophylactic anticoagulation in hospitalized GI malignancy patients and this decision should be reassessed daily. Empiric anticoagulation should be considered in all patients when there is a high clinical suspicion of PE. The decision to initiate therapeutic anticoagulation for acute pulmonary embolism in patients with intracranial metastases requires a risk-benefit analysis given the risk of intracranial hemorrhage. Although less commonly employed for submassive PE, mechanical thrombectomy affords rapid symptomatic relief and may be appropriate for patients at high risk of cardiopulmonary decompensation. An expedited evaluation and treatment of acute pulmonary embolism in high-risk patients by a multidisciplinary team is required to prevent significant morbidity and mortality.
